# NS3 epitope-decorated nanoparticles produced in bacteria trigger potent T cell immunity against hepatitis C virus

**DOI:** 10.7555/JBR.39.20250197

**Published:** 2026-03-19

**Authors:** Victor V. Kuprianov, Lyudmila I. Nikolaeva, Maya D. Stuchinskaya, Аnna A. Zykova, Nikolai V. Ravin

**Affiliations:** 1 Institute of Bioengineering, Research Center of Biotechnology of the Russian Academy of Sciences, Moscow 119071, Russia; 2 Department of Molecular Virology and Antiviral Drugs, Ivanovsky Institute of Virology, Gamaleya National Research Center for Epidemiology and Microbiology, Ministry of Health of the Russian Federation, Moscow 123098, Russia

**Keywords:** hepatitis C virus, NS3 epitope, nanoparticle, self-assembling peptide, vaccine

## Abstract

The highly conserved human leukocyte antigen-A2 (HLA-A2)-restricted epitope NS3-1073 represents a promising candidate for a therapeutic vaccine against hepatitis C virus (HCV). In this study, we engineered a set of fusion proteins based on the artificial self-assembling peptide (SAP), which were expressed in *Escherichia coli* and spontaneously self-assembled into nanosized particles displaying HCV epitopes, including NS3-1073. To enhance immunogenicity, we incorporated the T helper epitope PADRE into the construct. Alpha-helical linkers were introduced between SAP and the epitopes to facilitate proper protein folding. Notably, a helical linker with a high supercoiling propensity enabled soluble expression of the fusion protein containing both the NS3-1073 and PADRE epitopes, allowing purification of the *in vivo*-formed nanoparticles by metal affinity chromatography. Human dendritic cells derived from peripheral blood monocytes showed robust activation in response to the fusion proteins and preferentially stimulated T lymphocytes toward a Th1-biased immune response. In mice, immunization with nanoparticles carrying NS3-1073 induced splenocyte proliferation in response to *in vitro* stimulation with a mixture of NS3 peptides. These results demonstrate that recombinant nanoparticle-based carriers presenting the NS3-1073 epitope can be produced in bacterial systems and hold strong potential as a foundation for a therapeutic HCV vaccine.

## Introduction

Hepatitis C virus (HCV) is one of the most common pathogens causing chronic liver diseases globally. Approximately two million people become infected with HCV annually^[1]^. After the acute phase of infection, chronic hepatitis C develops in 70%–80% of cases, a condition that can gradually progress to cirrhosis and hepatocellular carcinoma^[2]^. New direct-acting antiviral drugs aimed at functionally important HCV proteins have increased the effectiveness of antiviral therapy, but do not guarantee complete eradication of the virus and are associated with side effects^[3]^. Therefore, the development of a therapeutic vaccine designed to achieve complete elimination of HCV from the body remains a relevant goal.

Current approaches to developing a therapeutic HCV vaccine include DNA and vector vaccines, recombinant proteins, and virus-like particles^[4]^. A new direction in the development of epitope vaccines is the use of self-assembling peptides (SAPs) to generate nanoparticles carrying target peptide antigens^[5–6]^. Such nanoparticles can enhance and focus the immune response to specific epitopes of selected proteins and can be used to develop vaccines. Therefore, in the current study, we used SAPs as carriers of HCV epitopes.

It has been established that the complicated mixture of distinct but related genomes known as a quasispecies constitutes the viral population in a hepatitis C patient^[7]^. This property of HCV plays a significant role in the formation of persistence, suppression of innate and adaptive immune responses, and promotion of the transition from acute to chronic infection^[8]^. To date, eight genotypes and several dozen subtypes of HCV have been reported^[9]^. A key challenge in the development of a therapeutic vaccine against HCV, as with other viruses with high variability, is the formation of a strong immune response to conserved immunodominant epitopes.

It has been demonstrated that patients with a broader and stronger T-cell response have a higher chance of recovering from acute hepatitis C^[10–12]^. However, in most cases, the T-cell response is insufficient, leading to chronic infection. Under these conditions, patients exhibit weakened HCV-specific T-cell responses^[13–15]^. The T-cell response in patients recovering from acute HCV infection is frequently directed toward epitopes of the viral protein NS3, which is a serine protease that specifically cleaves the viral polyprotein into individual proteins^[16]^. The formation of an immune response to the NS3-1073 epitope (amino acids 1073–1082 of the HCV polyprotein) is often correlated with recovery from acute HCV infection. Moreover, NS3-1073 epitope-specific CD8^+^ T cells persist for up to two decades after spontaneous recovery from hepatitis C^[17–19]^. Consequently, this epitope may be a promising candidate for inclusion in a therapeutic vaccine. Recently, the NS3-1073 peptide was used as a component of the IC41 vaccine, which has undergone evaluation in Phase Ⅰ and Phase Ⅱ clinical trials for therapeutic vaccination of patients with chronic HCV^[20–22]^. Analysis of the IC41 peptide vaccine indicated that NS3-1073 was an immunodominant CD8^+^ epitope, whereas immune responses to other CD8^+^ epitopes presented in the IC41 vaccine were insignificant in most vaccinated individuals, including both patients and healthy volunteers^[20–22]^. Therefore, we selected the NS3-1073 epitope for use in the design of nanoparticles and evaluated their immunogenicity as a prototype therapeutic vaccine against HCV.

Dendritic cells (DCs) are the key antigen-presenting cells responsible for orchestrating the immune response^[23–24]^. Therefore, evaluating DC activation by recombinant proteins can help to select the optimal design for a candidate therapeutic vaccine against hepatitis C. Additionally, activated DCs themselves can be used as a vaccine^[25–26]^.

In the current study, we constructed and characterized recombinant SAP-based proteins with helical linkers that can form nanoparticles displaying epitopes of the nonstructural protein NS3. Furthermore, we evaluated their immunogenicity and ability to activate DCs, thereby establishing a foundation for the development of a therapeutic vaccine against hepatitis C.

## Materials and methods

### Reagents

Isopropyl β-D-1-thiogalactopyranoside (IPTG; Cat. #I5502-1G), imidazole (Cat. #56748), mitomycin C (Cat. #M4287), the CCK-8 kit (Cat. #96992), and lipopolysaccharide from *Escherichia coli* (*E. coli*) O111:B4 (Cat. #L4391) were obtained from Sigma-Aldrich (Hamburg, Germany). Lysozyme (Cat. #L-040-1) was purchased from Gold Biotechnology (St. Louis, MO, USA); DNase E (Cat. #EK007S) from Evrogen (Moscow, Russia); Ni-NTA Agarose (Cat. #30230) from Qiagen (Hilden, Germany); guanidine hydrochloride (Cat. #Y0010) from Suzhou Yacoo Science (Suzhou, Jiangsu, China); PepTivator peptides "HCV 1b NS3" (Cat. #130-096-783) from Miltenyi Biotec (Bergisch Gladbach, Germany); Ficoll (Cat. #Р053), phytohemagglutinin (Cat. #М023), and RPMI-1640 medium with alanyl-glutamine (Cat. #С363п) from PanEco (Moscow, Russia); urea (Cat. #Am-0378-05) from VWR International LLC (Radnor, PA, USA); granulocyte-macrophage colony-stimulating factor (GM-CSF; Cat. #PSG030-05) and interleukin (IL)-4 (Cat. #PSG040-10) from SciStoreLab (Moscow, Russia); the test systems Interferon gamma (IFN-γ)-IFA-BEST (Cat. #А-8752), IL-2-IFA-BEST (Cat. #А-8772), and IL-4-IFA-BEST (Cat. #А-8754) from Vector-Best (Koltsovo, Russia); 100× antibiotic-antimycotic solution (Cat. #AAS-B) and gamma-irradiated fetal bovine serum (Cat. #FBS-GI-12A) from Capricorn Scientific (Ebsdorfergrund, Germany); and the Human CD14^+^ Cell Separation Kit (RUO) (Cat. #K1204-10) from RWD Life Science (Shenzhen, Guangdong, China).

### Bacterial strains

*E. coli* strains DLT1270 and DH5α from the laboratory collection were employed for gene cloning and the construction of expression vectors. *E. coli* DLT1270 was used for expression.

### Construction of plasmids

The nucleotide sequences encoding the designed proteins were synthesized by Evrogen (Moscow, Russia) and cloned into the expression vector pQE30 at the SacI and EcoRV restriction sites. Starting from the N-terminus, the recombinant proteins consisted of the following peptides: a hexahistidine tag (His), SAP^[5]^, helical linker (Sp or Spn), T-helper epitope PADRE (P, sequence: AKFVAAWTLKAAA)^[27]^, and epitopes of HCV genotype 1b nonstructural proteins (the HCV polyprotein 1b, GenBank accession no. AAC15724.1), including NS3: peptides FLATCINGVCWTVY (amino acids 1069–1082) and LLCPSGHVV (amino acids 1169–1177); NS4a: SGKPAIIPDREVLYQEFDEMEEC (amino acids 1689–1711); and NS5a: VLTDFKTWL (amino acids 1987–1995), LLPRLPGV (amino acids 1999–2006), VAAEEYVEIT (amino acids 2085–2094), and VILDSFEPLRAEEDEREVSVPAE (amino acids 2251–2273)^[18]^. Dilysine linkers were added between the epitopes to facilitate processing of the epitopes by DCs and their presentation^[28]^. The selection of amino acid sequences of linkers with different supercoiling propensities was based on literature data^[29]^ and the PCOILS program, available at https://toolkit.tuebingen.mpg.de/tools/pcoils^[30]^.

Synthetic genes were constructed for the expression of the following proteins: His-SAP-Sp-P-NS3, MRGSHHHHHHGSACELDMELRELQETLAALQDVRELLRQQVKQITFLKCLLMGGRLLCRLEELERRLEELERRLEELERRDLLAEAAAKEAAAKEAAAKEAAAKEAAAKAAADLAKFVAAWTLKAAADLKKFLATCINGVCWTVYKKLLCPSGHVVKKD (17792.62 Da); His-SAP-Spn-P-NS3, MRGSHHHHHHGSACELDMELRELQETLAALQDVRELLRQQVKQITFLKCLLMGGRLLCRLEELERRLEELERRLEELERRDLEEAAEEKKEEAAEEKKEEAAEEKKEEAAEEDLAKFVAAWTLKAAADLKFLATCINGVCWTVYKKLLCPSGHVVKKD (18317.85 Da); His-SAP-Spn-P-NS3,5a,4a, MRGSHHHHHHGSACELDMELRELQETLAALQDVRELLRQQVKQITFLKCLLMGGRLLCRLEELERRLEELERRLEELERRDLEEAAEEKKEEAAEEKKEEAAEEKKEEAAEEDLAKFVAAWTLKAAADLKKFLATCINGVCWTVYKKLLCPSGHVVKKDLKKVLTDFKTWLKKLLPRLPGVKKVAAEEYVEVTKKVILDSFEPLRAEEDEREVSVPAEILKKDIIIIGSRVDRSGKPAIIPDREVLYQEFDEMEEC (29640.02 Da); His-P-NS3,5a,4a, MRGSHHHHHHGSACELAKFVAAWTLKAAADLKKFLATCINGVCWTVYKKLLCPSGHVVKKDLKKVLTDFKTWLKKLLPRLPGVKKVAAEEYVEVTKKVILDSFEPLRAEEDEREVSVPAEILKKDIIIIGSRVDRSGKPAIIPDREVLYQEFDEMEEC (16245.1 Da).

### Protein expression and purification

Genes encoding the designed proteins were cloned into the pQE30 vector. Fusion proteins containing N-terminal hexahistidine sequences were expressed in *E. coli* DLT1270 cells. The cultures were grown in 2xYT medium supplemented with 100 μg/mL ampicillin.

It is known that a decrease in the concentration of antibiotics in the growth medium during induction can increase the production of recombinant protein^[31]^. Therefore, we used a modified induction protocol that enhanced the expression of the desired recombinant proteins. Specifically, an overnight culture grown in 2xYT medium with 100 μg/mL ampicillin was diluted to an OD_600_ of 0.7 using approximately five volumes of fresh 2xYT medium without ampicillin. After adding 0.5 mmol/L IPTG, the culture was incubated at 28 ℃ for an additional 18 h.

To purify the recombinant proteins, cells from a 50 mL culture were collected by centrifugation at 13000 *g* for 5 min, and then resuspended in 1 mL of sodium phosphate-tris (SPT) buffer (10 mmol/L Na_2_HPO_4_, 10 mmol/L Tris-HCl, pH 8.0) with 20 mmol/L imidazole and 500 mmol/L NaCl. Cells were lysed by incubation with lysozyme (1 mg/mL) at room temperature for 15 min, followed by freezing and thawing, sonication, and incubation with DNase E (0.01 mg/mL) at 37 ℃ for 10 min. The lysate was then centrifuged at 13000 *g* for 10 min.

If the expressed protein was soluble and detected in the supernatant, it was purified using Ni-NTA agarose (Qiagen), equilibrated with SPT buffer containing 20 mmol/L imidazole and 500 mmol/L NaCl (the His-SAP-Spn-P-NS3 protein). The protein was eluted in SPT buffer containing 1 mol/L imidazole. If the protein was detected in the pellet (His-SAP-Sp-P-NS3, His-SAP-Spn-P-NS3,5a,4a, and His-P-NS3,5a,4a), after cell lysis, it was dissolved in SPT buffer with 7 mol/L guanidine hydrochloride, 20 mmol/L imidazole, and 0.5 mol/L NaCl, sonicated, and kept at room temperature for 10–15 min. Then, the solution was centrifuged at 13000 *g* for 10 min. The resulting supernatant was collected and incubated at room temperature for 30 min with Ni-sorbent equilibrated with the same buffer. The Ni-sorbent was washed with SPT buffer containing 7 mol/L guanidine hydrochloride and 30 mmol/L imidazole, and then with SPT buffer containing 9 mol/L urea and 30 mmol/L imidazole. Finally, the purified protein was eluted in SPT buffer containing 1 mol/L imidazole and 4.5 mol/L urea. The eluates were dialyzed at 8 ℃ against phosphate-buffered saline (PBS; pH 7.3) with 1 mol/L urea and subsequently dialyzed twice against PBS at a volume ratio of 1∶500. The results of the expression and purification of recombinant proteins were analyzed using sodium dodecyl sulfate polyacrylamide gel electrophoresis (SDS-PAGE).

### Transmission electron microscopy (TEM) analysis

TEM analysis was performed using a JEM-1400 device (JEOL, Tokyo, Japan). The purified proteins were applied to carbon-collodion-coated copper grids (TED PELLA, Redding, CA, USA) for 5 min. Then, the excess samples were removed. The grids were washed two times with water. Using a 1% uranyl acetate solution, the negative staining technique was applied. Finally, the grids were stained with 1% (w/v) uranyl acetate and analyzed by TEM.

### Determination of nanoparticle size by dynamic light scattering

A Zetasizer Nano S90 particle size analyzer (Malvern, Worcestershire, United Kingdom) with 12 mm square polystyrene cuvettes (DTS0012) was used to evaluate particle size by dynamic light scattering at 25 ℃. The purified protein preparation with a concentration of 1 mg/mL after dialysis against PBS was diluted tenfold with water and analyzed for the presence of nanoparticles according to the manufacturer's instructions.

### Obtaining dendritic cells from human peripheral blood monocytes and their activation with recombinant proteins

The study was conducted with the voluntary, informed consent of five healthy volunteers. Human monocytes were isolated from the volunteers' peripheral blood leukocytes by centrifugation in a Ficoll-Hypaque density gradient followed by positive selection of CD14^+^ cells on magnetic beads. Unadsorbed lymphocytes were collected and stored in 10% DMSO with fetal bovine serum at −80 ℃. To obtain DCs, monocytes were cultured in RPMI-1640 medium supplemented with 10% fetal bovine serum, GM-CSF at 1000 U/mL, and interleukin-4 at 500 U/mL for five days. For the maturation of DCs, they were further incubated for two days with lipopolysaccharide from *E. coli* at a concentration of 1 μg/mL. Mature DCs were then incubated in RPMI-1640 medium containing 2% autologous human serum and 10 μg/mL of recombinant protein for three days. Subsequently, the DCs were treated with mitomycin C at a concentration of 30 μg/mL at 37 ℃ for 40 min and washed four times with PBS. After this, they were co-cultured with autologous lymphocytes.

The study on obtaining dendritic cells from human peripheral blood monocytes was approved by the Ethics Committee of Infectious Diseases Clinical Hospital No. 1 (Protocol No. 8 dated 28.12.2022).

### Analysis of T-lymphocyte proliferative activity

For activation, T lymphocytes were co-cultured with mitomycin C-treated, stimulated DCs at a ratio of 10∶1 for five days. Then, a mixture of PepTivator peptides "HCV 1b NS3" at a concentration of 1 μg/mL was added and incubated for an additional 24 h. As a positive control, lymphocytes were stimulated with 10 μg/mL phytohemagglutinin. Proliferative activity was assessed using the CCK-8 kit. The stimulation index was calculated as the ratio of the optical density of stimulated cells to that of unstimulated cells. Each reaction was performed in triplicate.

### Determination of IFN-γ, IL-2, and IL-4 in the culture medium

Similar to the assessment of proliferative activity, T lymphocytes were co-cultured with activated DCs for five days. The levels of IFN-γ, IL-2, and IL-4 in the culture medium were assessed on the third day after the secondary stimulation of lymphocytes with a mixture of PepTivator peptides "HCV 1b NS3" using the test systems IFN-γ-IFA-BEST, Interleukin-2-IFA-BEST, and Interleukin-4-IFA-BEST, respectively. Cytokine levels reflect the efficacy of the specific T-cell immune response in relation to the NS3-1073 epitope. Each test was performed in triplicate.

### Immunization of mice

BALB/c mice (18–20 g, 6–8 weeks old) were immunized subcutaneously in the anterior part of the back three times at 2-week intervals. Each injection contained 50 μg of recombinant protein without adjuvant. Control mice were injected with PBS. For analysis, the spleens were harvested from the mice on the seventh day following the last immunization. Each experimental group consisted of five mice. The study on mice was conducted according to the European Convention for the Protection of Vertebrate Animals and was approved by the Ethics Committee of the Gamaleya National Research Center (Protocol No. 83 dated 11/28/2024).

### Proliferative activity of spleen cells

Spleen cells were isolated from immunized mice using a mouse spleen dissociation kit (Cat. #130-095-926, Miltenyi Biotec) according to the manufacturer's instructions and seeded at 1 × 10^5^/well. Splenocytes were stimulated by incubation with 1 μg/mL of the PepTivator® HCV1b NS3 multiepitope peptide mixture (Miltenyi Biotec) for 54 h. Splenocyte proliferation was analyzed using the CCK-8 kit according to the manufacturer's instructions. The stimulation index was calculated as the ratio of the optical density determined for the stimulated lymphocyte culture to that of the unstimulated culture. A stimulation index greater than 2 was considered indicative of a positive proliferative response^[28]^.

### Statistical analysis

All assays were performed in triplicate. Data were expressed as the mean ± standard error of the mean. Student's *t*-test was used to compare the data. *P*-values less than 0.05 were considered statistically significant.

## Results

### Design and production of recombinant proteins

**Figure 1:**
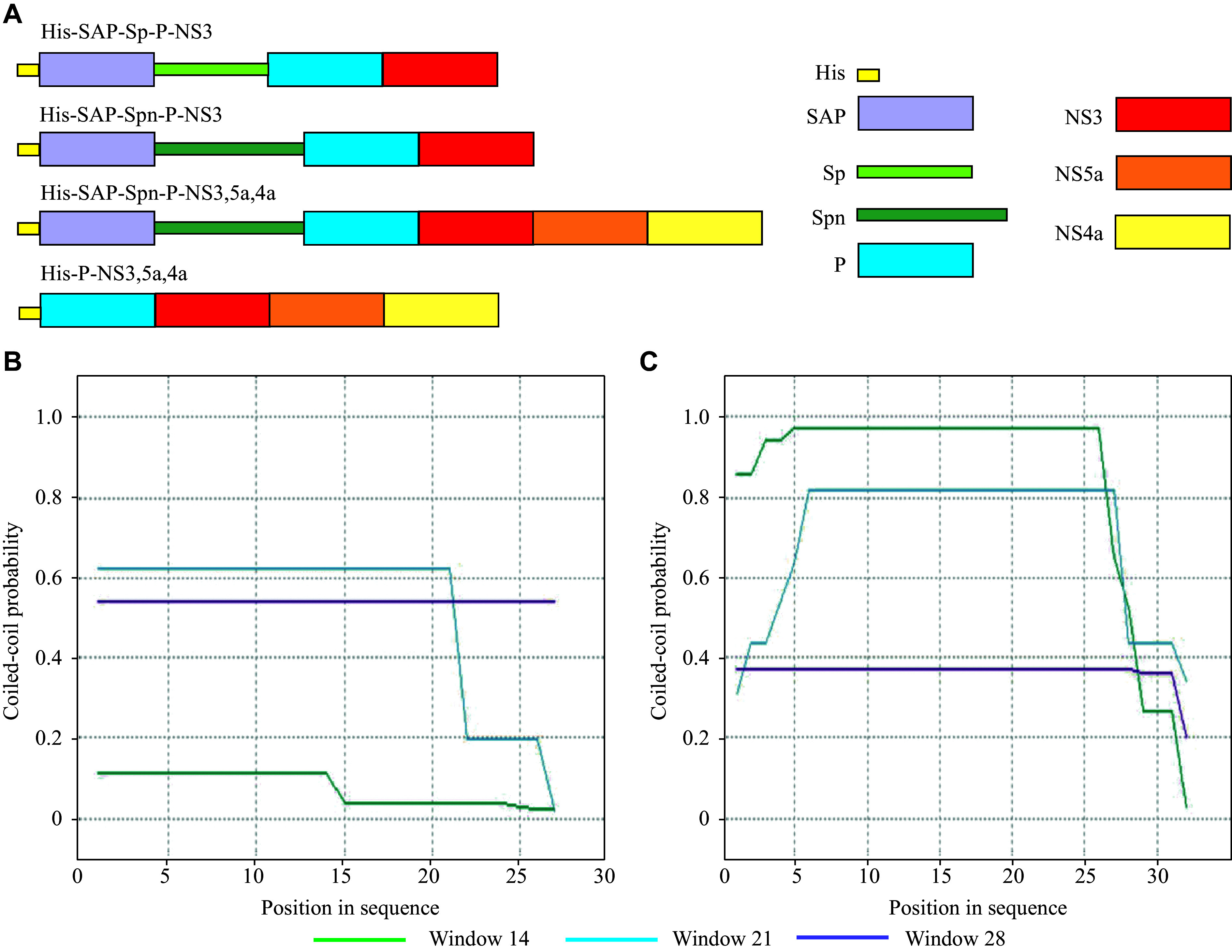
Structure of recombinant proteins. A: Schematic representation of recombinant fusion proteins. B and C: Analysis of the amino acid sequence of the Sp (B) and Spn (C) linkers using the PCOILS program. The probability of formation of a coiled-coil structure is shown. Abbreviations: His, hexahistidine tag; NS3, epitopes from nonstructural protein 3; NS4a, epitopes from nonstructural protein 4A; NS5a, epitopes from nonstructural protein 5A; P, Th epitope PADRE; SAP, self-assembling peptide; Sp, helical linker; Spn, modified helical linker.

Studies have demonstrated that the NS3-derived epitopes FLATCINGVCWTVYK and KLLCPSGHVV, corresponding to amino acids 1069–1082 and 1169–1177 in the HCV polyprotein 1b, respectively, can elicit specific T-cell responses^[18–19]^. Thus, we constructed recombinant proteins containing SAP and these NS3 epitopes (***Fig. 1A***). A universal Th epitope PADRE (AKFVAAWTLKAAA) was inserted between them to enhance immunogenicity^[27]^. Additionally, dilysine linkers were inserted between the epitopes to improve their processing and presentation by DCs^[28]^.

The presence of a helical linker between two genetically fused proteins helps preserve their functional properties^[29]^. Supercoiling refers to the ability of alpha helices in proteins to interact with each other and form intermolecular structures. We investigated the influence of different helical linkers on the formation of nanoparticles during the expression of recombinant proteins in *E. coli* by using linkers with varying superhelical (coiled-coil) properties. The original Sp linker (LEAAAKEAAAKEAAAKEAAAKEAAAKD)^[29]^ and the modified linker, designated Spn (LEEAAEEKKEEAAEEKKEEAAEEKKEEAAEED), were designed. Sequence analysis of these linkers using the PCOILS program^[30]^ showed that Spn has a greater propensity for supercoiling than Sp (***Fig. 1B*** and ***1C***).

We designed three recombinant proteins based on SAP, each incorporating a C-terminal helical linker (Sp or Spn), the Th epitope PADRE (P), and T cell epitopes derived from HCV nonstructural proteins. The constructs were designated as His-SAP-Sp-P-NS3, His-SAP-Spn-P-NS3, and His-SAP-Spn-P-NS3,5a,4a. The recombinant protein His-P-NS3,5a,4a, which lacked SAP and was not capable of forming nanoparticles, was used as a control to assess the effect of SAP and linkers on immunogenicity (***Fig. 1A***).

**Figure 2:**
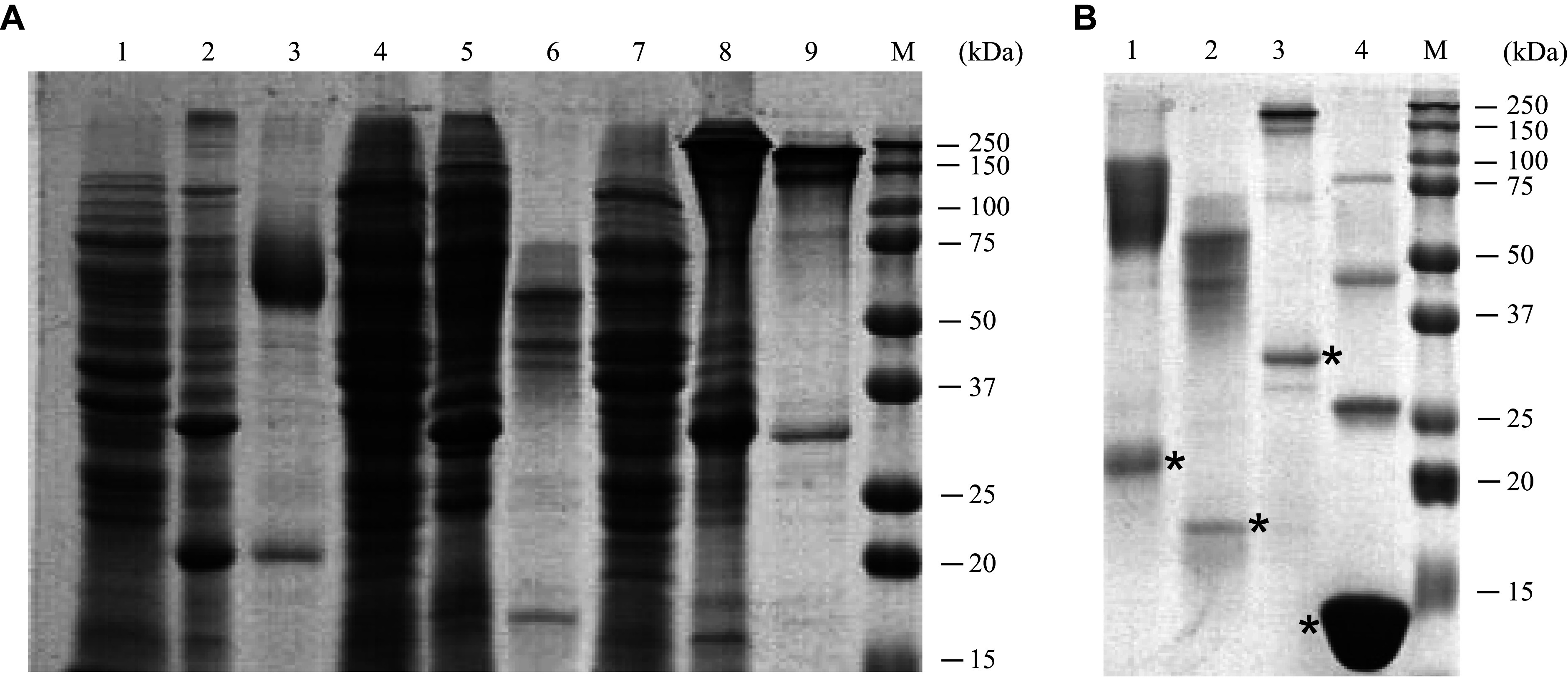
Sodium dodecyl sulfate polyacrylamide gel electrophoresis (SDS-PAGE) analysis of recombinant proteins. A: Expression and purification of recombinant proteins. Lanes 1–3, His-SAP-Sp-P-NS3 (cell lysate, insoluble fraction, purified protein); lanes 4–6, His-SAP-Spn-P-NS3 (cell lysate, insoluble fraction, purified protein); lanes 7–9, His-SAP-Spn-P-NS3,5a,4a (cell lysate, insoluble fraction, purified protein); lane 10, molecular weight marker. B: Analysis of purified proteins with alkylation. The positions of the monomers are marked with an asterisk. Lane 1, His-SAP-Sp-P-NS3; lane 2, His-SAP-Spn-P-NS3; lane 3, His-SAP-Spn-P-NS3,5a,4a; lane 4, His-P-NS3,5a,4a.

Recombinant proteins were produced in *E. coli* cells. Analysis of protein expression indicated that His-SAP-Sp-P-NS3 was insoluble, whereas His-SAP-Spn-P-NS3 was predominantly soluble and could be purified using metal chelate chromatography under native conditions (***Fig. 2A***). Furthermore, His-SAP-Sp-P-NS3 protein isolated under denaturing conditions and renatured at concentrations above 1 mg/mL aggregated post-dialysis and formed a precipitate, while the protein with the Spn linker was stable in solution.

The obtained recombinant proteins each contained five cysteine residues, which caused their aggregation into oligomers, as observed during electrophoresis in SDS-PAGE. To identify protein monomers, purified protein preparations were alkylated with iodoacetamide (***Fig. 2B***). Alkylation reduced the number of oligomers and increased the amount of monomers. The monomers of His-SAP-Sp-P-NS3 and His-SAP-Spn-P-NS3 exhibited apparent molecular masses of approximately 20 kDa and 17 kDa, respectively. The addition of epitopes from other nonstructural proteins of HCV to the C-terminus resulted in the expression of the recombinant protein His-SAP-Spn-P-NS3,5a,4a in an insoluble form.

### Characterization of nanoscale structures by electron microscopy and dynamic light scattering

**Figure 3:**
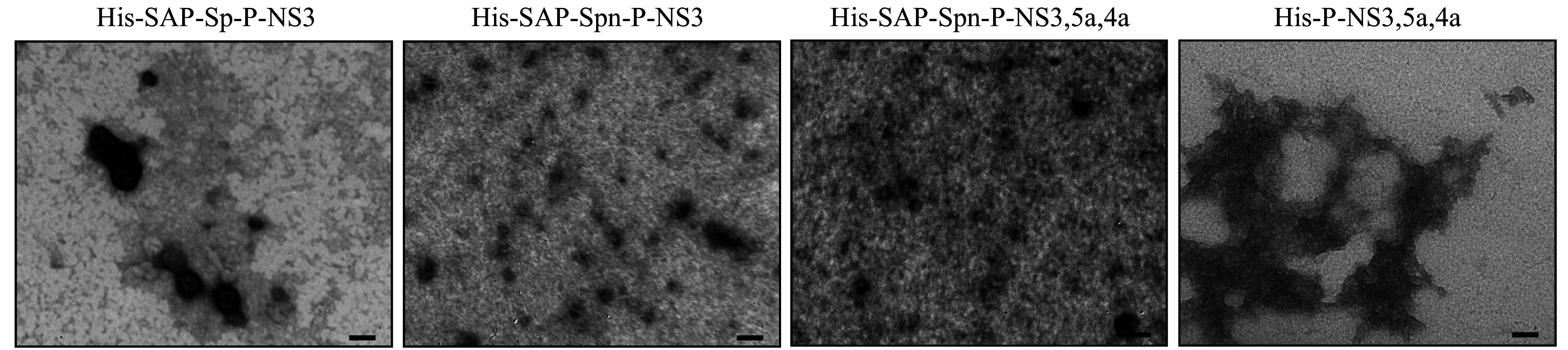
Transmission electron microscopy analysis of purified proteins. Scale bar, 50 nm.

Electron microscopy of purified protein samples showed the presence of nanoparticles for all proteins containing SAP sequences (***Fig. 3***). In the case of the His-SAP-Sp-P-NS3 protein after refolding, nanoparticles were primarily observed as aggregates, whereas fewer aggregates were observed for the His-SAP-Spn-P-NS3 protein purified under native conditions. For the His-SAP-Spn-P-NS3,5a,4a protein after refolding, individual particles were also observed. The particle size was 30–40 nm in all samples. However, the His-P-NS3,5a,4a protein did not form nanoparticles, but aggregates up to several microns in size were detected.

**Figure 4:**
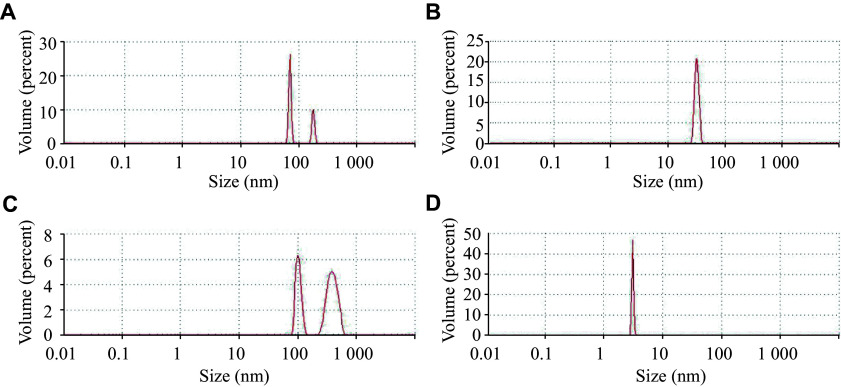
Nanoparticle size distribution in purified protein samples revealed by dynamic light scattering analysis. Proteins: His-SAP-Sp-P-NS3 (A), His-SAP-Spn-P-NS3 (B), His-SAP-Spn-P-NS3,5a,4a (C), and His-P-NS3,5a,4a (D).

Nanoparticle size was further analyzed by dynamic light scattering using a Zetasizer Nano S90 particle size analyzer. The results showed that the His-SAP-Sp-P-NS3 sample contained two types of nanoparticles, measuring 178 nm and 71 nm in diameter (***Fig. 4A***); the His-SAP-Spn-P-NS3 sample contained only 32 nm particles (***Fig. 4B***); the His-SAP-Spn-P-NS3,5a,4a sample contained 394 nm and 103 nm nanoparticles (***Fig. 4C***), whereas the His-P-NS3,5a,4a contained only small aggregates approximately 3 nm in size (***Fig. 4D***).

### Immunogenic characteristics of recombinant proteins

To compare the immunogenic properties of the obtained recombinant fusion proteins, human DCs were activated *in vitro* with these proteins and subsequently used to stimulate autologous lymphocytes. The stimulated lymphocytes were then given a secondary stimulation with a mixture of peptides from the NS3 protein of HCV to assess the specific immune response to NS3 epitopes. After the secondary stimulation, the proliferative activity of lymphocytes was measured, and cytokine (*i.e.*, IFN-γ, IL-2, and IL-4) levels in the culture medium were determined.

**Figure 5:**
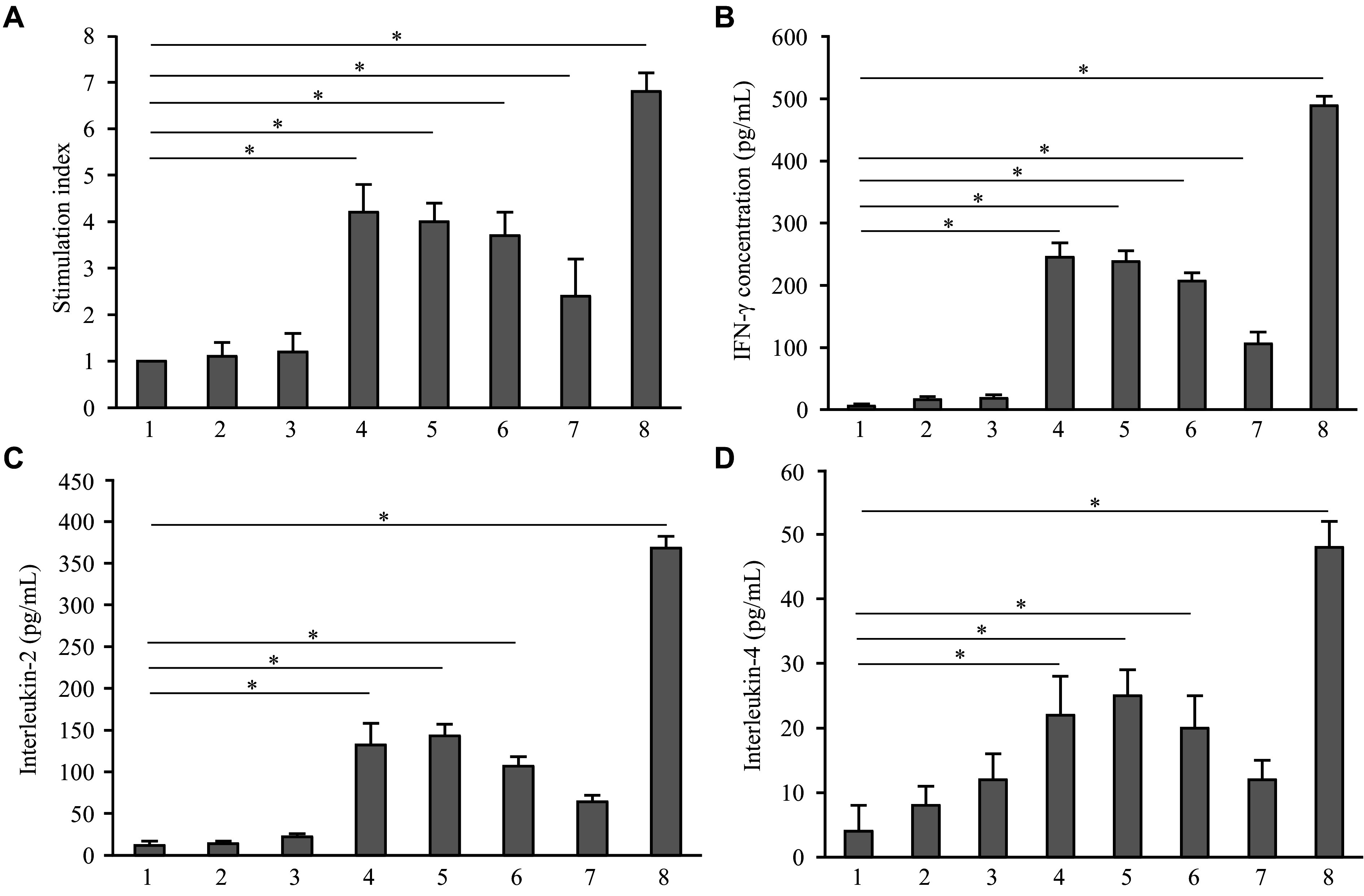
Analysis of immune response *in vitro*. Human dendritic cells were activated by recombinant proteins *in vitro* and then used to stimulate autologous lymphocytes. Stimulated lymphocytes were analyzed after a secondary stimulation with a mixture of peptides from the NS3 protein of the hepatitis C virus. A: Proliferative activity of lymphocytes. B–D: Interferon-γ (IFN-γ; B), interleukin (IL)-2 (C), and IL-4 (D) levels in the culture medium. Lanes: 1, PBS (negative control); 2, His-SAP-Sp; 3, His-SAP-Spn; 4, His-SAP-Sp-P-NS3; 5, His-SAP-Spn-P-NS3; 6, His-SAP-Spn-P-NS3,5a,4a; 7, His-P-NS3,5a,4a; 8, phytohemagglutinin (PHA, positive control). Each test was performed in triplicate. Data are shown as mean ± standard error of the mean. Normally distributed data were compared using the Student's *t*-test. ^*^P < 0.05.

The comparison of lymphocyte proliferative activity showed that recombinant proteins with SAP carrying NS3 epitopes activated DCs more effectively than those without SAP. The proliferation index for nanoparticles containing Sp and Spn linkers was nearly the same, with values of 4.2 (± 0.6) and 4.0 (± 0.4), respectively. However, His-SAP-Spn-P-NS3,5a,4a had a slightly lower proliferation index of 3.7 (± 0.5), compared with particles containing only NS3 epitopes. The protein without SAP had the lowest proliferative activity index of 2.4 (± 0.8) (***Fig. 5A***).

Measurement of key cytokines revealed that nanoparticles His-SAP-Sp-P-NS3 and His-SAP-Spn-P-NS3 exhibited the highest immunogenicity. The cytokine concentrations were as follows: 245 (± 23) pg/mL and 238 (± 22) pg/mL for IFN-γ, 110 (± 26) pg/mL and 143 (± 14) pg/mL for IL-2, and 22 (± 6) pg/mL and 25 (± 4) pg/mL for IL-4, respectively. Notably, nanoparticles His-SAP-Spn-P-NS3,5a,4a with additional epitopes were less immunogenic compared with those carrying only NS3 epitopes, and the content of interleukins was lower: 207 (± 13) pg/mL for IFN-γ, 107 (± 11) pg/mL for IL-2, and 20 (± 5) pg/mL for IL-4. For the recombinant protein without SAP, the cytokine content in the culture medium was the lowest: 106 (± 19) pg/mL for IFN-γ, 64 (± 8) pg/mL for IL-2, and 12 (± 3) pg/mL for IL-4 (***Fig. 5B***–***5D***).

**Figure 6:**
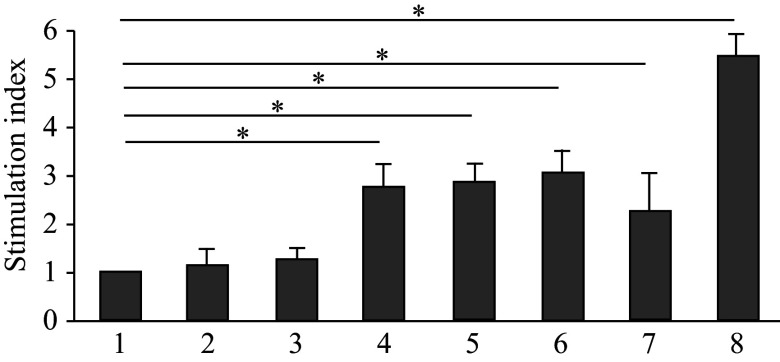
Proliferative activity of splenocytes of immunized mice after stimulation with NS3 peptides. Lanes: 1, PBS (negative control); 2, His-SAP-Sp; 3, His-SAP-Spn; 4, His-SAP-Sp-P-NS3; 5, His-SAP-Spn-P-NS3; 6, His-SAP-Spn-P-NS3,5a,4а; 7, His-P-NS3,5a,4a; 8, phytohemagglutinin (PHA, positive control). Each test was performed in triplicate. Data are shown as mean ± standard error of the mean. Normally distributed data were compared using the Student's *t*-test. ^*^P < 0.05.

Mice were immunized to study the immunogenicity of the recombinant proteins. The results showed that the recombinant protein preparations were non-toxic, as the mice did not lose weight during the immunization process. The specific T cell response in the immunized mice was determined by measuring the proliferative activity of splenocytes in response to *in vitro* stimulation with a mixture of NS3 peptides. It was found that the stimulation index, characterizing proliferative activity, was 2.8–3.1 for SAP-containing proteins and 2.3 for proteins without SAP (***Fig. 6***).

Thus, we demonstrated that a recombinant protein containing SAP with a helical linker Spn and carrying the PADRE and NS3 epitopes formed nanoparticles in a soluble form, which successfully induced a T-cell immune response in human peripheral blood cells *in vitro* and in mice *in vivo*.

## Discussion

HCV is a positive-strand RNA virus characterized by a high degree of genetic diversity because its RNA polymerase is prone to errors. The viral population in patients with hepatitis C consists of a quasispecies^[32]^. Therefore, the use of conserved amino acid sequences of viral proteins is relevant for the formation of effective immunity. The ability to induce functional HCV-specific T cells is considered a key property of therapeutic HCV vaccines. The HCV-specific epitope NS3-1073 is one of the most studied epitopes in HCV. The immune response against NS3-1073 is often associated with recovery from acute HCV infection^[10–12,33]^. Thus, generating a potent T-cell response to this epitope is a promising avenue for developing a therapeutic HCV vaccine.

Studies on hybrid recombinant proteins have shown that the use of rigid helical linkers preserves the functional properties of the original proteins and facilitates their production in bacterial cells^[29]^. We therefore evaluated the effect of helical linkers on nanoparticle assembly based on their supercoiling ability. The PCOILS program was used to select the amino acid sequence of the linkers^[30]^. The helical linker (Sp) originally described by Arai *et al*^[29]^ had a slight tendency to supercoil. We designed a modified linker (Spn) that showed a greater tendency to supercoil.

Recombinant SAP-based proteins containing Sp and Spn linkers, which separate HCV and PADRE epitopes from SAP, were designed. These proteins were successfully produced in *E. coli* cells. The protein with the Sp linker was expressed in an insoluble form, and nanoparticles could only be obtained by *in vitro* refolding of the protein purified under denaturing conditions. In contrast, the protein with the Spn linker was produced mostly in a soluble form and could be purified under native conditions. The purified His-SAP-Spn-P-NS3 preparations contained nanoparticles that were apparently formed *in vivo* within *E. coli* cells.

His-SAP-Sp-P-NS3 and His-SAP-Spn-P-NS3 particles exhibited comparable immunogenicity both in experiments using immunocompetent cells from human peripheral blood and during mouse immunization. Both particles stimulated Th1 and Th2 pathways, with a predominance of the Th1 response. Unfortunately, we lacked the opportunity to use advanced methods, such as flow cytometry, to detect the characteristics of lymphocytes after stimulation. Nevertheless, enzyme immunoassay-based methods enabled us to obtain reliable data on the immunogenicity of recombinant proteins, which was the primary objective of our study.

The use of the recombinant His-SAP-Spn-P-NS3 protein, instead of His-SAP-Sp-P-NS3, simplifies the technology for producing immunogenic nanoparticles that carry epitopes of the nonstructural protein NS3. This advancement paves the way for the future development of a therapeutic HCV vaccine in the form of protein nanoparticles derived from a bacterial expression system. However, due to the absence of an appropriate animal model, we were unable to assess the efficacy of the recombinant proteins as a therapeutic vaccine *in vivo*. Consequently, we used human DCs to evaluate our recombinant proteins that form nanoparticles.

Several studies on the development of an HCV vaccine based on DCs have recently been reported^[32–35]^. DCs are the most effective antigen-presenting cells and specialize in capturing and converting proteins into peptides that form a complex with MHC molecules, which are exposed on the surface of DCs and recognized by T cells^[34–35]^. Therefore, studying the ability of recombinant proteins based on SAP to activate DCs *in vitro* allows for a direct assessment of the prospects for using a particular recombinant protein as a therapeutic vaccine candidate for hepatitis C, both in the form of nanoparticles and in the form of DCs activated by them.

Currently, there are many approaches to developing therapeutic vaccines against chronic hepatitis C. These vaccines aim to stimulate an antigen-specific immune response. Peptide vaccines, however, have not been sufficiently effective because they weakly stimulate T-cell immunity. Vaccines based on DNA and RNA vectors, such as plasmids, mRNA, and defective viral particles, have proven their effectiveness, but their use is associated with unpredictable consequences due to the possibility of integrating genetic material into the patient's genome^[36]^.

Recombinant proteins containing HLA-specific epitopes of viral proteins and adjuvant components, such as self-assembling peptides that promote the formation of nanoparticles, have greater specific immunogenicity compared with short peptides. Therefore, we consider the development of therapeutic vaccines based on recombinant proteins with an internal adjuvant to be a safe and promising direction.
